# Prognostic value of CD8 + PD-1+ immune infiltrates and PDCD1 gene expression in triple negative breast cancer

**DOI:** 10.1186/s40425-019-0499-y

**Published:** 2019-02-06

**Authors:** Joe Yeong, Jeffrey Chun Tatt Lim, Bernett Lee, Huihua Li, Clara Chong Hui Ong, Aye Aye Thike, Wei Hseun Yeap, Yi Yang, Ansel Yi Herh Lim, Timothy Kwang Yong Tay, Jin Liu, Siew-Cheng Wong, Jinmiao Chen, Elaine Hsuen Lim, Jabed Iqbal, Rebecca Dent, Evan W. Newell, Puay Hoon Tan

**Affiliations:** 1 Division of Pathology, Singapore General Hospital, 20 College Road, Academia, Level 7, Singapore, 169856 Singapore; 2 Singapore Immunology Network (SIgN), Agency of Science, Technology and Research (A*STAR), 8A, Biomedical Grove, Immunos, Singapore, 138648 Singapore; 3 Division of Medicine, Singapore General Hospital, Singapore, Singapore; 4 Duke-NUS Medical School, Singapore, Singapore; 5 Centre for Quantitative Medicine, Duke-NUS Medical School, Singapore, Singapore; 6 Shanghai University of Finance and Economics, Shanghai, China; 7 Yong Loo Lin School of Medicine, National University of Singapore, Singapore, Singapore; 8 National Cancer Centre Singapore, 11 Hospital Drive, Singapore, 169610 Singapore

**Keywords:** TNBC, PD-1, PD-L1, Immune checkpoint, IFNG

## Abstract

The role of programmed cell death protein-1 (PD-1)/programmed cell death ligand 1 (PD-L1) in triple negative breast cancer (TNBC) remains to be fully understood. In this study, we investigated the role of PD-1 as a prognostic marker for TNBC in an Asian cohort (*n* = 269). Samples from patients with TNBC were labeled with antibodies against PD-L1 and PD-1, and subjected to NanoString assays to measure the expression of immune-related genes. Associations between disease-free survival (DFS), overall survival (OS) and biomarker expression were investigated. Multivariate analysis showed that tumors with high PD-1^+^ immune infiltrates harbored significantly increased DFS, and this increase was significant even after controlling for clinicopathological parameters (HR 0.95; *P* = 0.030). In addition, the density of cells expressing both CD8 and PD-1, but not the density of CD8^−^PD-1^+^ immune infiltrates, was associated with improved DFS. Notably, this prognostic significance was independent of clinicopathological parameters and the densities of total CD8^+^ cell (HR 0.43, *P* = 0.011). At the transcriptional level, high expression of *PDCD1* within the tumor was significantly associated with improved DFS (HR 0.38; *P* = 0.027). In line with these findings, high expression of *IFNG* (HR 0.38; *P* = 0.001) and IFN signaling genes (HR 0.46; *p* = 0.027) was also associated with favorable DFS. Inclusion of PD-1 immune infiltrates and *PDCD1* gene expression added significant prognostic value for DFS (ΔLRχ^2^ = 6.35; *P* = 0.041) and OS (ΔLRχ^2^ = 9.53; *P* = 0.008), beyond that provided by classical clinicopathological variables. Thus, PD-1 mRNA and protein expression status represent a promising, independent indicator of prognosis in TNBC.

**Electronic supplementary material:**

The online version of this article (10.1186/s40425-019-0499-y) contains supplementary material, which is available to authorized users.

## Introduction

Triple negative breast cancer (TNBC) accounts for 9–17% of all breast cancer diagnoses [1–3] and is defined by the absence of estrogen receptor (ER), progesterone receptor (PR) and c-erbB2 (HER2) expression. Although TNBC is histopathologically heterogeneous, these tumors share common clinical challenges. Patients frequently present with advanced disease, suffer a high incidence of metastasis and recurrence, and have significantly poorer prognosis than patients whose tumors express the aforementioned receptors [4–6]. Oncologic management options are limited due to the lack of therapeutic targets. As a result, almost 50% of patients with TNBC succumb to the disease within 5 years of diagnosis [5].

The recent success of immunotherapy targeting programmed cell death protein-1 (PD-1)/programmed cell death ligand 1 (PD-L1) in other cancers, such as non-small cell lung cancer and melanoma, has yet to be achieved in TNBC regardless of which monoclonal antibodies (including pembrolizumab, durvalumab, atezolizumab and avelumab) are used [7–15]. However, TNBC harbors relatively high numbers of tumor-infiltrating lymphocytes (TILs) [16–18], frequently expresses higher levels of PD-L1 [19–21] and has an elevated tumor mutational burden [22, 23] compared with other breast cancer subtypes. Therefore, in order to identify novel targets for immunotherapy and those individuals most likely to respond to treatment, further elucidation of the TNBC immune microenvironment is necessary.

Immune cells are known to be a determining factor in tumor initiation, progression and metastasis [24, 25], but understanding precisely which cell types act to promote or prevent disease, and under what circumstances, have proven challenging. For example, during the immunoediting process [26, 27], TILs and the immune system serve different roles in the three **E** phases. These include the initial phase of cancer cell **E**limination, an **E**quilibrium phase during which the surviving cancer cells undergo immune-mediated dormancy and, ultimately, **E**scape from immunosurveillance in the final phase. In breast cancer, high TIL levels are associated with reduced survival in patients with ER^+^ breast tumors, but this same feature is associated with a significantly increased survival time in TNBC [4, 28–30]. The mere presence of TILs is therefore an insufficient predictor of their influence. For this reason, there remains an urgent need to characterize the TIL compartment more thoroughly, particularly in the context of concurrent loss of hormone receptors and HER2 expression. In addition to T and B cells, natural killer cells and macrophages may also infiltrate tumors, but the role served by PD-1^+^ T cells is of particular clinical interest at present [31–39].

PD-1 expression is known to be associated with T cell exhaustion. In a general setting without immunotherapy, high PD-1^+^CD8^+^ T cell levels are associated with a poor prognosis in a range of cancers, including liver cancer, pancreatic cancer, early breast cancer and head and neck cancers [40–45]. However, the notion that all PD-1^+^ immune cells are “exhausted” and, therefore, that they should be promoting pro-tumor immunity, may be an oversimplification. For example, a recent study demonstrated that tumor-infiltrating T cells in breast cancer expressed PD-1, but not other markers associated with exhaustion, and that these cells produced similar levels of pro-inflammatory cytokines to effector T cells [46]. Whether PD-1 is a marker of exhaustion, activation or both remains controversial, and PD-1 expression may only demonstrate that an immune cell has been recently stimulated, and is therefore antigen-experienced [47–51]. Furthermore, PD-1 expression on TILs, especially relative to tumor cell PD-L1 expression, is not a good predictive marker for PD-1/PD-L1 checkpoint blockade immunotherapy [40, 47, 52–55], and the function of these cells in many types of cancer, including TNBC, is not fully understood.

Considering the evident importance of the PD-1/PD-L1 pathway in determining clinical outcomes in multiple cancers, and the dearth of knowledge surrounding the involvement of this pathway in TNBCs, our group used multimodal methodologies, including conventional pathology techniques, multiplex immunofluorescent (mIF) staining and NanoString to retrospectively evaluate PD-1^+^ total immune infiltrates, the CD8^+^PD-1^+^ subset, PD-L1 protein expression, and transcript levels of *CD274, PDCD1* and *IFNG.* We subsequently identified the factors among these that were associated with clinical outcomes.

## Materials and methods

### Patients and tumors

A total of 269 archival formalin-fixed, paraffin-embedded (FFPE) TNBC specimens from patients diagnosed between January 2003 and December 2013 at the Department of Anatomical Pathology, Division of Pathology, Singapore General Hospital, were analyzed. All samples were obtained before patients underwent adjuvant chemo- or radiotherapy. Clinicopathological parameters, including patient age, tumor size, histologic growth pattern, grade and subtype, associated ductal carcinoma in situ, lymphovascular invasion and axillary lymph node status, are reviewed in Additional file 1: Table S1. The age of patients ranged from 28 to 89 years (median, 55 years) while length of follow-up ranged from 1 to 213 months (mean, 101 months; median, 97 months); with recurrence and death occurring in 65 (24%) and 45 (17%) of these women, respectively. Tumors were typed, staged and graded according to the World Health Organization, American Society of Clinical Oncology-College of American Pathologists (ASCO-CAP) guidelines [47]. The Centralized Institutional Review Board of SingHealth provided ethical approval for the use of patient materials in this study (CIRB ref.: 2013/664/F and 2015/2199).

### Tissue microarray (TMA) construction

Tumor regions for TMA construction were selected based on pathological assessment, which identified samples where > 50% of the sample area was tumor tissue. For each sample, two or three representative tumor cores of 1 mm diameter were transferred from donor FFPE tissue blocks to recipient TMA blocks using an MTA-1 Manual Tissue Arrayer (Beecher Instruments, Inc., Sun Prairie, WI, USA). TMAs were constructed as previously described [6].

### Immunohistochemical analysis of TMAs

TMA sections (4 μm thick) were labeled with antibodies against PD-1, PD-L1, CD8, ER, PR and HER2 (Additional file 1: Table S2). We also labeled tumor sections with antibodies against epidermal growth factor receptor (EGFR), cytokeratin (CK) 14 and CK high molecular weight (clone 34βE12) to identify TNBC with a basal-like phenotype, according to previously published protocols [6, 48]. Appropriate positive and negative controls were included. Scoring of antibody-labeled sections was performed for nuclear ER and PR, membranous HER2, EGFR and PD-L1, cytoplasmic CK14, 34βE12 and PD-1, and membranous and/or cytoplasmic CD8 positivity. To generate the scores, images of labeled slides were captured using a ScanScope XT device (Aperio Technologies; Leica Microsystems GmbH, Wetzlar, Germany) or an IntelliSite Ultra-Fast Scanner (Philips Research, Eindhoven, Netherlands) prior to examination by two pathologists blinded to clinicopathological and survival information. ASCO-CAP guidelines were used to define positivity cut-offs for the tumors as follows: a positive ER/PR result was defined as the presence of at least 1% of tumor cell nuclei displaying unequivocal staining of any intensity, and for HER2, tumor positivity was defined as > 10% of tumor cells exhibiting 3+ membrane staining [49]. Ambiguous HER2 cases were tested and confirmed by fluorescence in situ hybridization based on the ASCO/CAP guidelines [50, 51]. CK14, EGFR and 34βE12 positivity was also determined in accordance to the aforementioned 1% cut-off [6, 48].

Tumor PD-L1 expression was confirmed when staining of the tumor cell membranes (of any intensity) was observed at prespecified expression threshold levels of 1% or higher in a TMA core including at least 100 tumor cells that could be evaluated [52–55].

The number of PD-1^+^ immune infiltrates was counted for every 1 mm diameter TMA core, following previously described methods [19, 45, 56–58]. Samples were then grouped into “high” and “low” according to whether the PD-1^+^ immune infiltrate count was above the median or equal to/below the median [59–61].

### Multiplex immunofluorescence (mIF)

Multiplex immunofluorescence (mIF) was performed using an Opal Multiplex fIHC kit (PerkinElmer, Inc., Waltham, MA, USA) as previously described by our group and other studies [45, 61–71], on FFPE tissue sections processed according to the standard immunohistochemistry protocol described above. Slides were labeled with primary antibodies against PD-1 and CD8, followed by appropriate secondary antibodies (as presented in Additional file 1: Table S2), before application of the fluorophore-conjugated tyramide signal amplification buffer (PerkinElmer, Inc., Waltham, MA, USA). DAPI was used as a nuclear counterstain. Images were acquired using a Vectra 3 pathology imaging system microscope (PerkinElmer, Inc.) and analyzed using inForm version 2.3 software (PerkinElmer, Inc.) [63, 72, 73].

CD8 was stained using Opal 540 (Catalog No. FP1494001KT) while PD-1 was stained by using Opal 620 (Catalog NO. FP1495001KT). The counterstain DAPI was from Catalog No. FP1490. They were purchased from PerkinElmer, Inc., Waltham, MA, USA.

### RNA extraction, NanoString measurement of *PDCD1* and *CD274* expression, and analysis

RNA was extracted from 8 unlabeled FFPE sections (10 μm thick) using an RNeasy FFPE kit (Qiagen GmbH, Hilden, Germany) on a QIAcube automated sample preparation system (Qiagen GmbH), and was quantified using an Agilent 2100 Bioanalyzer system (Agilent Technologies, Santa Clara, CA, USA). A total of 100 ng of functional RNA (> 300 nucleotides) was assayed on the nCounter MAX Analysis System (NanoString Technologies, Inc., Seattle, WA, USA). The NanoString counts were normalized using positive control probes and the housekeeping genes, as previously reported [61, 71]. The count data were then logarithmically transformed prior to further analysis. *P* < 0.05 was considered to indicate a statistically significant difference.

### Cell lines and flow cytometry

All human breast adenocarcinoma cell lines were a gift from Dr. Sandra Hubert (SIgN). BT20, HCC-38, HCC1806, MDA-MB-231, MDA-MB-453 and MDA-MB-468 were maintained in RPMI cultured with 10% (*v*/v) heat-inactivated fetal calf serum (HI-FCS, Gibco; Thermo Fisher Scientific, Inc., Waltham, MA, USA), 1% (v/v) penicillin-streptomycin (PS) at 37 °C and 5% CO_2_ in a cell culture incubator.

The antibodies to measure protein expression during flow cytometry using the above cell lines were α-PDL1 (Clone 29E.2A3, IgG2b, Cat No: 32970, BioLegend, San Diego, CA, USA), α-PDL2 (Clone 24F.10CL2, IgG2a, Cat No: 329608, BioLegend) and α-HLA-ABC (Clone W6/32, IgG2a, Cat No: 311413, BioLegend). Cell lines were trypsinised using PBS-EDTA. For flow cytometry, 0.5 million cells from each cell line were resuspended in homemade FACs buffer (1x PBS + 0.2 M EDTA + 20% (v/v) HI-FCS + 20% (v/v) human serum), incubated with various antibodies for 20 min at 4 °C and analyzed using FACSAria II with 4 lasers (BD Biosciences, San Jose, CA, USA).

### Gene heat map, validation, follow-up and statistical analysis

Follow-up data were obtained from medical records. Disease-free survival (DFS) and overall survival (OS) were defined as the time from diagnosis to recurrence or death/date of last follow-up, respectively. Statistical analysis was performed using SPSS 23.0 for Windows (IBM Corp., Armonk, NY, USA). The associations between clinicopathological parameters and the frequency of PD-1^+^ immune infiltrates and PD-L1^+^ tumor cells were tested using χ^2^ and Fisher’s exact tests. Survival outcomes were estimated using Kaplan-Meier analysis and groups were compared using log-rank statistics. Multivariate Cox regression was performed to evaluate the effect of PD-1 and PD-L1 status and the NanoString *PDCD1* and *CD274* counts on survival, after adjusting for clinicopathological parameters including patient age, tumor size, tumor grade and lymph node status. NanoString percentile thresholds for *PDCD1* and *CD274* were tested using log-rank statistics for OS, and the best percentile thresholds were used to define the *PDCD1* and *CD274* double-positive samples. Gene expression percentile thresholds for *PDCD1* and *CD274* were determined in the same fashion, using public data from METABRIC, and then used to define the *PDCD1* and *CD274* double-positive samples. Gene expression and survival data for METABRIC and The Cancer Genome Atlas (TCGA) were obtained from cBioPortal for validation purposes, after filtering for TNBC samples. Models were compared using the increment in the log-likelihood of the models (∆LRχ^2^) using a likelihood ratio test. *P* < 0.05 was considered to indicate a statistically significance difference.

## Results

### Patients with tumors harboring a high density of PD-1^+^ immune cells have improved clinical outcomes

**Fig. 1:**
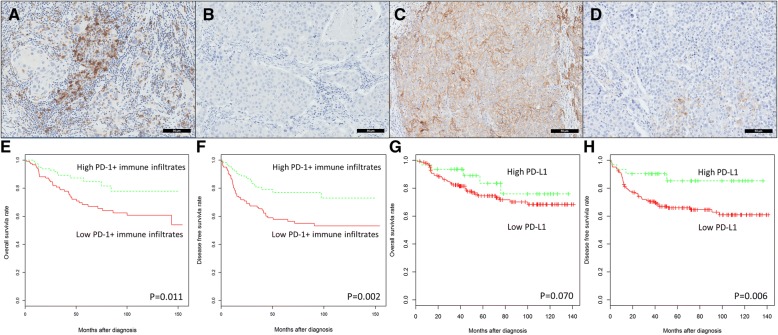
PD-1^+^ immune infiltrates and PD-L1 tumor cell expression in TNBC. Representative immunohistochemical staining showing **a** high and **b** low PD-1^+^ immune infiltrates; and **c** high and **d** low PD-L1 tumor cell expression in TNBC sections (magnification, 100x). High PD-1^+^ immune infiltrates and PD-L1 tumor cell expression are associated with improved survival in TNBC. Kaplan-Meier analysis of **e** OS and **f** DFS outcomes in women with high versus low densities of PD-1^+^ immune infiltrates. Kaplan-Meier analysis of **g** OS and **h** DFS outcomes in women with high versus low PD-L1 tumor cell expression

Tissue sections from TNBC were incubated with antibodies targeting PD-1 to allow identification of total PD-1^+^ immune infiltrates (Fig. 1a-d). The number of PD-1^+^ immune infiltrates was counted in every 1 mm diameter TMA core, following previously published methods [19, 45, 56–58]. Samples were then grouped according to whether their PD-1^+^ immune infiltrates counts were high (above the median), or low (equal to/below the median). Meanwhile, PD-L1 expression was quantified as a tumor proportion score, as previously described [52–55].

In the case of total PD1^+^ immune infiltrates, 46.6% of the TNBC samples were determined to have high levels of PD1^+^ immune infiltrates, and 26.5% of samples were determined to have high PD-L1 tumor cell protein expression (Additional file 1: Table S3). Univariate analysis of the clinicopathological features of high and low PD-1 immune infiltrates and PD-L1 tumor cell expression revealed that tumors with high levels of PD-1 immune infiltrates were significantly more likely to lack lymphovascular invasion (*P* = 0.034; Additional file 1: Table S1), which is a key feature reflecting tumor aggressiveness [74, 75].

We then investigated whether PD-1^+^ immune infiltrates in tumors had any effect on outcomes in patients with TNBC. As presented in Fig. 1e-h, Kaplan-Meier survival analysis revealed that TNBC patients in the “high PD-1^+^ immune infiltrates” group had significantly improved OS and DFS compared with those in the “low PD-1^+^ immune infiltrates” group (OS, *P* = 0.01; DFS, *P* = 0.002). Interestingly, Kaplan-Meier survival analysis also revealed that the “high PD-L1 tumor cell expression” group had improved DFS compared with the “low PD-L1 tumor cell expression group” (*P* = 0.006), while OS was not significantly different between the groups (*P* = 0.070).

Multivariate analysis further supported the association between a high density of PD-1^+^ immune infiltrates in TNBC and a significantly improved DFS (HR = 0.48; 95% CI 0.29–0.81; *P* = 0.005), and this effect was evident at every 1 cell increment of PD-1 immune infiltrate density (Table 1). In other words, every incremental 1 cell per 1 mm core was associated with improved DFS (HR = 0.95; 95% CI 0.93–1.00; *P* = 0.030). Multivariate analysis similarly demonstrated that high PD-L1 tumor cell expression was associated with improved OS (HR = 0.40; 95% CI 0.18–0.86; *P* = 0.020) and DFS (HR = 0.39; 95% CI 0.20–0.76; *P* = 0.006).

**Table 1:** Multivariate analysis of PD-1^+^ immune infiltrates and PD-L1 tumor cells with survival outcomes in patients with TNBC. Analysis was adjusted for tumor size, grade, age and lymph node status

Biomarkers	HR	95% CI	*P*-value
OS			
PD-1^+^ immune infiltrates High vs. low	0.61	0.33–1.13	0.110
PD-1^+^ immune infiltrates (every 1 cell increment)	0.96	0.91–1.01	0.080
PD-L1 expression High vs. low	0.40	0.18–0.86	0.020*
DFS			
PD-1^+^ immune infiltrates High vs. low	0.48	0.29–0.81	0.005*
PD-1^+^ immune infiltrates (every 1 cell increment)	0.95	0.93–1.00	0.030*
PD-L1 expression High vs. low	0.39	0.20–0.76	0.006*

*PD-1* programmed cell death protein-1, *PD-L1* programmed cell death ligand 1

*Statistically significant

**Fig. 2:**
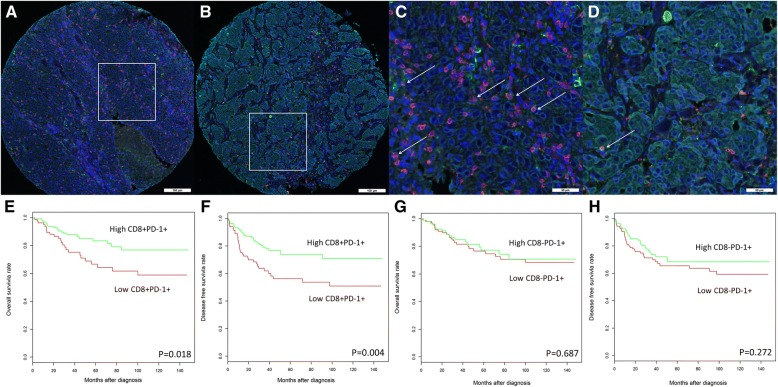
PD-1^+^ immune infiltrates have at least two subsets in relation to CD8 cells in TNBCs; the CD8^+^PD-1^+^ double positive subset and the CD8^−^PD-1^+^ subset. mIF labeled sections from two representative TNBC tissue samples show the PD-1^+^, CD8^+^, the CD8^+^PD-1^+^ double positive subset and the CD8^−^PD-1^+^ subset within the tumor microenvironment. mIF labeling for PD-1 (green), CD8 (red), Pan-cytokeratin (cyan) and DAPI (blue). **a** TNBC harbored high CD8^+^PD-1^+^ double positive subset. **b** TNBC harbored low CD8^+^PD-1^+^ double positive subset. **c** and **d** Higher magnification of the same region from images (**a** and **b**, respectively) shows CD8^+^PD-1^+^ double positive subset through double labeling of CD8 (red) and PD-1(green), indicated by white arrows. High levels of CD8^+^PD-1^+^ infiltrates, but not high levels of CD8^−^PD-1^+^ immune infiltrates, are associated with improved survival in TNBC. Kaplan-Meier analysis of **e** OS and **f** DFS outcomes in women with high versus low densities of CD8^+^PD-1^+^ double positive immune infiltrates. Kaplan-Meier analysis of **g** OS and **h** DFS outcomes in women with high versus low CD8^−^PD-1^+^ immune infiltrates

Furthermore, Opal mIF staining [45, 62–71] for PD-1 and CD8 was performed on TNBC sections, followed by image acquisition with a Vectra 3 pathology imaging system and image analysis with inForm software [63, 72, 73]. Notably, the immune subsets that expressed both CD8 and PD-1 (Fig. 2a-d) were associated with improved survival (Fig. 2e-f), but CD8^−^PD1^+^ immune infiltrates were not (Fig. 2a-d and g-h). Multivariate analysis revealed that the CD8^+^PD1^+^ double positive immune subset was an independent prognostic marker for improved DFS (HR = 0.45; 95% CI 0.28–0.80; *P* = 0.006), even when adjusted for both clinicopathological parameters and total CD8^+^ T cell counts (HR = 0.43; 95% CI 0.23–0.83; *P* = 0.011) (Table 2) the latter previously reported by our group, to highlight the prognostic influence of intratumoral CD8^+^ T cell density in TNBC [60].

**Table 2:** Multivariate analysis of CD8^+^PD-1^+^ double positive immune subsets with survival outcomes in patients with TNBC. Analysis was adjusted for tumor size, grade, age and lymph node status

Biomarkers	HR	95% CI	*P*-value
OS			
CD8^+^PD-1^+^ immune infiltrates High vs. low	0.56	0.29–1.06	0.073
CD8^+^PD-1^+^ immune infiltrates High vs. low (adjusted for tumor size, grade, age and lymph node status and CD8^+^ total cell count)	0.77	0.35–1.67	0.510
DFS			
CD8^+^PD-1^+^ immune infiltrates High vs. low	0.47	0.28–0.80	0.006*
CD8^+^PD-1^+^ immune infiltrates High vs. low (adjusted for tumor size, grade, age and lymph node status and CD8^+^ total cell count)	0.43	0.23–0.83	0.011*

*PD-1* programmed cell death protein-1, *PD-L1* programmed cell death ligand 1

*Statistically significant

### Higher PD-1 and PD-L1 gene expression is associated with an improved clinical outcome in TNBC

There was evidence of a significant positive correlation between the densities of PD-1^+^ immune infiltrates and PD-L1 tumor cell expression (*P* < 0.0001; *R* = 0.303) (Additional file 1: Table S4). Meanwhile, correlations between protein and mRNA levels of PD-L1 and PD-1 were clear (PD-L1 vs. *CD274*, *P* < 0.0001, *R* = 0.411; PD-1 vs. *PDCD1*, *P* < 0.0001, *R* = 0.276; Additional file 1: Tables S4 and S5, respectively).

We proceeded to examine the link between the expression levels of *PDCD1* (encoding PD-1), *CD274* (encoding PD-L1), and TNBC prognosis. We utilized a NanoString assay [76, 77] to measure PD-1^+^ immune infiltrates and PD-L1 tumor cell expression at the transcriptional level in the TNBC samples, and then compared transcript abundance with survival time. As presented in Table 3, every incremental unit of either *PDCD1* and *CD274* was associated with improved OS (*PDCD1,* HR 0.02, 95% CI 0.00–0.36, *P* = 0.007; *CD274,* HR 0.12, 95% CI 0.02–0.81, *P* = 0.030) and DFS (*PDCD1,* HR 0.08, 95% CI 0.01–0.83, *P* = 0.034; *CD274,* HR 0.19, 95% CI 0.04–0.97, *P* = 0.045) even following adjustment for tumor size, grade, age and lymph node status.

**Table 3:** Multivariate analysis of *PDCD1*, *CD274, IFNG and IFN signaling genes* RNA expression survival outcomes in patients with TNBC. Analysis was adjusted for tumor size, grade, age and lymph node status

Biomarkers	HR	95% CI	*P*-value
OS			
*PDCD1* expression (every 1 unit increment)	0.02	0.00–0.36	0.007*
*CD274* expression (every 1 unit increment)	0.12	0.02–0.81	0.030*
*IFNG* expression High vs. low	0.38	0.21–0.72	0.003*
Interferon signaling gene expression High vs. low	0.59	0.29–1.17	0.132
DFS			
*PDCD1* expression (every 1 unit increment)	0.08	0.01–0.83	0.034*
*CD274* expression (every 1 unit increment)	0.19	0.04–0.97	0.045*
*IFNG* expression High vs. low	0.38	0.22–0.68	0.001*
Interferon signaling gene expression High vs. low	0.46	0.23–0.92	0.027*

*Statistically significant

These results were confirmed using *PDCD1* and *CD274* gene expression data from a publicly-available database (EGAS00001001753 from the European Genome-Phenome Archive), which revealed a significant association between increased *PDCD1* and *CD274* expression and DFS (*PDCD1* HR 0.38, 95% CI 0.15–0.94, *P* = 0.027; *CD274* HR 0.63, 95% CI 0.42–0.96, *P* = 0.026) but not OS (*PDCD1* HR 0.55, 95% CI 0.26–1.12, *P* = 0.086; *CD274* HR 0.77, 95% CI 0.55–1.08, *P* = 0.121) in 320 cases of TNBC (Additional file 1: Table S6).

**Fig. 3:**
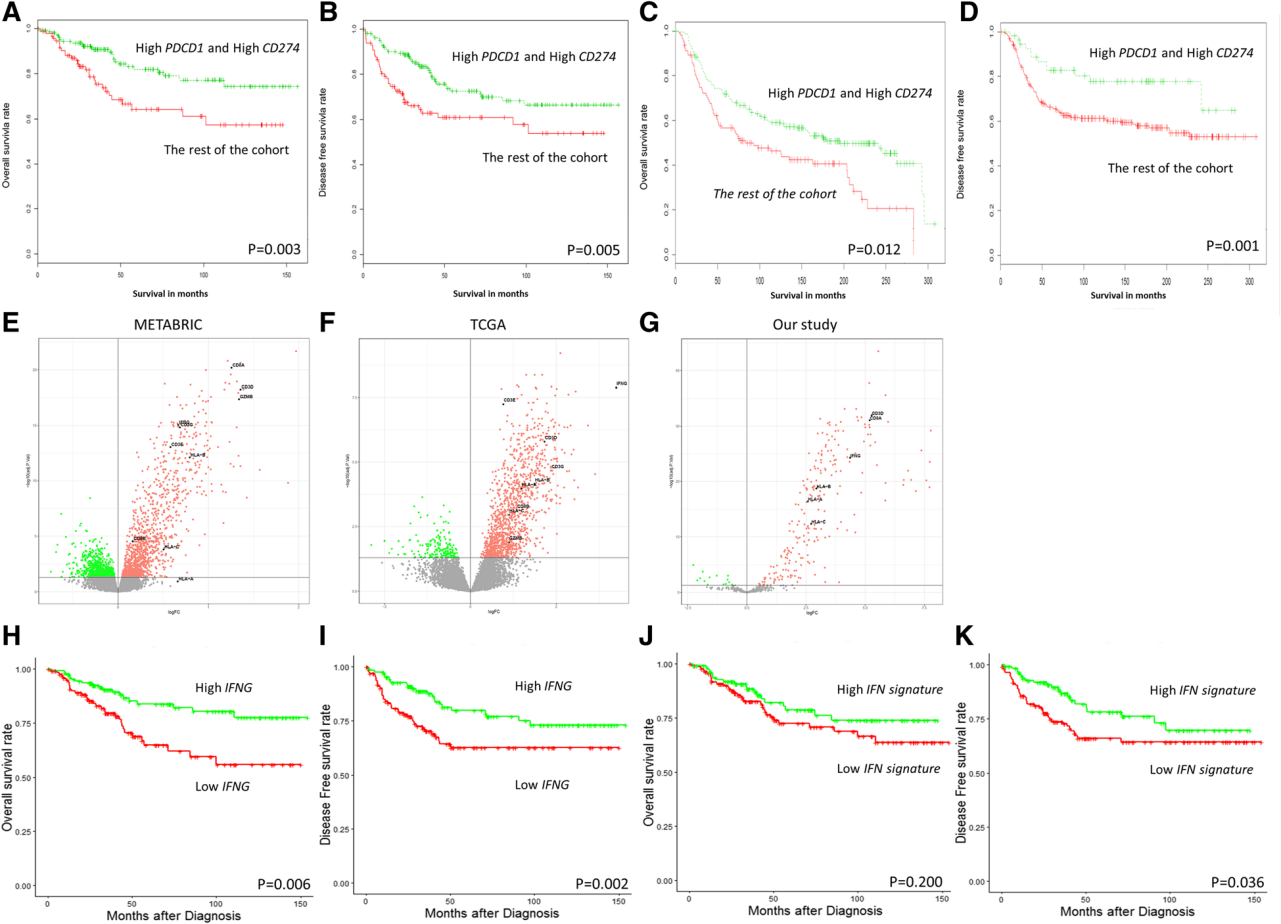
High *PDCD1* and high *CD274* mRNA expression is associated with improved survival in TNBC. Kaplan-Meier analysis of **a** OS and **b** DFS outcomes in women with high *PDCD1* and high *CD274* expression, compared with the rest of the cases in the cohort. Both high *PDCD1* and high *CD274* are associated with improved survival in the METABRIC public TNBC dataset. From the publicly available TNBC dataset, Kaplan-Meier analysis of **c** OS and **d** DFS outcomes in women with high *PDCD1* and high *CD274* compared with the rest of the cases in the cohort (*n* = 320). High *PDCD1* and high *CD274* are both associated with increased levels of T cells and MHC-I genes in TNBC, in both public dataset and our NanoString gene expression data. In the group with high *PDCD1* and high *CD274* expression, a significant increase of certain key DEGs was observed. These genes are associated with T cells and MHC-I molecules, and include *CD8A, CD8 CD3D, CD3E, CD3G, HLA-A, HLA-B* and *HLA-C* from **e** METABRIC and **f** TCGA, two publically available datasets, and **g** our NanoString data. Kaplan-Meier analysis of **h** OS and **i** DFS outcomes in women with high *IFNG* expression, compared with low *IFNG* cases in the cohort. Kaplan-Meier analysis of **j** OS and **k** DFS outcomes in women with high *IFN* associated signature 5 gene expression based on the canonical pathway “Interferon signaling”, compared with the rest of the cases in the cohort

Given the strong association between *PDCD1* and *CD274* expression levels and patient survival, a group of TNBC patient samples which harbored high *PDCD1* and high *CD274* was defined. The expression levels of both genes were higher than the optimal percentile threshold in these patients, as determined using OS. With this definition, as expected, the prognostic value of these two markers in combination was still present, with the high *PDCD1* and high *CD274* group being associated with improved OS (*P* = 0.003) and DFS (*P* = 0.005), compared with the rest of the patients (Fig. 3a-b).

We then investigated the overall gene expression profiles of tumors from high *PDCD1* and high *CD274* patients, compared with the rest of the patients, to look for additional molecular correlates that could explain differences between the tumors of these patients. A customized panel of a NanoString assay was utilized to measure the expression of a panel of 499 immune and cancer-associated genes in the TNBC cohort [59, 78–84]. One way ANOVA followed by post hoc t-tests revealed that 77 genes were significantly differentially expressed, with a fold-change of > 2 fold, between TNBC cases that harbored both high *PDCD1* and high *CD274* expression and the rest of the cases (Additional file 1: Figure S1). Ingenuity Pathway Analysis (IPA) was used to decipher the biological functions of the 77 differentially-expressed genes (DEGs). Core analysis in IPA identified significant functional enrichment in the expression of genes associated with canonical pathways: “communication between innate and adaptive immunity” (*P* = 0.003) and “Interferon signaling” (*P* = 0.008). Furthermore, IPA upstream regulator analysis also revealed that interferon gamma (*IFNG*) was enriched (*P* = 0.001).

The prognostic value of the *CD274* and *PDCD1* combination and the DEGs was validated by the aforementioned publicly-available database (EGAS00001001753 from the European Genome-Phenome Archive [85]; Fig. 3c-d). Interestingly, as presented in the volcano plots (Fig. 3e-g), CD3 and CD8 genes, along with *IFNG* and major histocompatibility complex class I (MHC-I) genes (*HLA-A*, *HLA-B* and *HLA-C),* were among the highly expressed DEGs in the high *CD274* and high *PDCD1* expression group. This finding was consistent across three cohorts, which included public data such as METABRIC [85] and TCGA [22] (Additional file 1: Figure S2) which was obtained from cBioPortal [86, 87], as well as our own cohort.

The increase of PD-L1 and the reduction of MHC-I have long been considered one of the key events and mechanisms underlying immune escape [88–91]. However, our group found that *CD274* gene expression was significantly associated with *HLA-A*, *HLA-B* and *HLA-C* expression, as shown in Additional file 1: Table S7. This result was further supported through the use of human TNBC cell lines, with flow cytometry staining performed to determine the expression of PD-L1 and MHC-I (*R* = 0.89; Additional file 1: Figure S3).

Based on the IPA canonical pathway analysis and the upstream regulator analysis showing that *IFNG* is enriched based on the DEGs and because *IFNG* represents a critical cytokine in immunoediting [92–94] and is functionally linked to PD-L1 and PD-1 [95], we further investigated the prognostic role of *IFNG* in this cohort. As presented in Fig. 3h-i, high *IFNG* expression is associated with favorable DFS (*P* = 0.006) and OS (*P* = 0.002). As shown in Table 3, multivariate analysis further confirmed this result following adjustment of clinicopathological parameters (DFS HR 0.38, 95% CI 0.22–0.68; *P* = 0.0009; OS HR 0.38, 95% CI 0.21–0.72; *P* = 0.0027). Furthermore, 5 genes which are demonstrated in the IPA canonical pathway analysis as “Interferon signaling”, and their expression levels were examined in this TNBC cohort as shown in Additional file 1: Figure S4., Unsupervised hierarchical analysis revealed the existence of two distinct clusters of TNBC (Additional file 1: Figure S4): cluster 1 (green) contained TNBC with higher *IFN signaling gene* expression, with these patients exhibiting significantly improved DFS (*P* = 0.036) as shown in Fig. 3j-k, but not OS, compared with the low *IFN-*signaling-gene-expressing cluster 2 (red). This finding is further confirmed with multivariate analysis (HR 0.46 95% CI 0.23–0.92; *P* = 0.027) as shown in Table 3.

### PD-1^+^ immune infiltrates, PD-L1 tumor cell expression and the expression of *CD274* and *PDCD1* add significant prognostic power to classical clinicopathological parameters

To further demonstrate the prognostic power of the PD-1/PD-L1-associated measures reported in the present study, we examined the impact of incorporating their effects into survival outcome analysis with a panel of typical clinicopathological features (patient age, tumor grade, tumor size and lymph node status). As presented in Table 4, the addition of PD-L1^+^ tumor cells to clinicopathological features significantly increased the prognostic value for DFS (∆LRχ^2^ = 5.22; *P* = 0.022), and OS (∆LRχ^2^ = 3.95; *P* = 0.047). On the other hand, the addition of PD-1^+^ immune infiltrate density to clinicopathological features significantly increased the prognostic value for DFS (∆LRχ^2^ = 4.18; *P* = 0.028), but not OS, compared with clinicopathological features alone. Meanwhile, the inclusion of *PDCD1* gene expression increased the prognostic value for both DFS (∆LRχ^2^ = 4.12; *P* = 0.043) and OS (∆LRχ^2^ = 6.55; *P* = 0.011). Of the multiple proteins, genes and combinations, PD-1^+^ immune infiltrates combined with *PDCD1* gene expression conferred the best added prognostic value for both DFS (∆LRχ^2^ = 6.35; *P* = 0.042) and OS (∆LRχ^2^ = 9.53; *P* = 0.009). Notably, for DFS alone, the CD8^+^PD-1^+^ double positive immune subset offered the best prognostic value (∆LRχ^2^ = 7.53; *P* = 0.006). In addition, *IFNG* alone showed good prognostic value for both DFS (∆LRχ^2^ = 7.50; *P* = 0.006) and OS (∆LRχ^2^ = 5.29; *P* = 0.022).

**Table 4:** Table showing the change in the log-likelihood of the models with added prognostic terms. Statistical significance of the change was determined by a likelihood ratio test

Variables	DFS		OS	
	∆LRχ^2^	*P*-value	∆LRχ^2^	*P*-value
CP + PD-1 vs. CP	4.83	0.0280*	2.95	0.0856
CP + PD-L1 vs. CP	5.22	0.0224*	3.95	0.0469*
CP + *CD274* vs. CP	3.66	0.0559	4.32	0.0378*
CP + *PDCD1* vs. CP	4.12	0.0425*	6.55	0.0105*
CP + *CD274* + *PDCD1* vs. CP	4.49	0.1057	6.12	0.0469*
CP + PD-L1 + *CD274* vs. CP	4.11	0.1278	7.02	0.0299*
CP + PD-1^+^ + *PDCD1* vs. CP	6.35	0.0417*	9.53	0.0085*
CP + CD8^+^PD-1^+^ vs. CP	7.53	0.0061*	3.16	0.0753
CP + *IFNG* vs. CP	7.50	0.0062*	5.29	0.0214*

*CP* Clinicopathological parameters (patient age, tumor grade, tumor size and lymph node status), *PD-1*^*+*^ PD-1^+^ immune infiltrates (every 1 cell increment), *PD-L1* PD-L1 tumor cell expression, *LR* Likelihood Ratio, *CD8*^*+*^*PD-1*^*+*^ CD8^+^PD-1^+^ T cells

*Statistically significant

## Discussion

In the present study, we demonstrated that patients bearing TNBC with high PD-1^+^ immune infiltrates and high PD-L1 tumor expression were likely to experience significantly improved clinical outcomes, and this was validated both at the transcriptional level as well as through a separate cohort, using publicly accessible data. Furthermore, our results demonstrated that it is the CD8^+^ PD-1^+^ double-positive immune subset specifically that offers prognostic value, while the CD8^-^PD-1^+^ immune subset does not. Also, scoring of whole sections showed statistically significant correlation with that on TMAs for PD-1^+^ immune infiltrates. (Additional file 1: Figure S5A and B).

To the best of our knowledge, this report is the first to highlight the prognostic value of PD-1^+^, as well as CD8^+^PD-1^+^ immune infiltrates, through multivariate analysis and mIF, and to highlight the significant correlation with PD-L1 expression in tumor cells in TNBC. Similar associations between high PD-L1 expression in tumor cells and improved prognosis have been reported in several recent studies concerning TNBC [55, 96–99] as well as hormone receptor-positive breast cancers [100–104]. However, some reports have suggested that PD-L1 may be a prognostic marker of breast cancer in general, but one that is associated with worse prognosis [21, 105–108]. This would suggest that for non-TNBC, PD-L1 expression may not be a particularly robust prognostic marker. One possibility is that the prognostic impact of PD-L1 is dependent on the hormone receptor status of the tumor, suggesting either direct or indirect roles of hormone receptors in the regulation of tumor immunity; a topic that warrants further investigation.

In addition to the hormone receptor status of the tumor, infiltrating immune cells and MHC-I may be key to the prognostic significance of the PD-L1/PD-1 pathway. Therefore, our investigations focused primarily on PD-1^+^ immune infiltrates. By comparing the prognostic models with a likelihood ratio test, using the increment in the log-likelihood of the models (∆LRχ^2^), we demonstrated that a combination of PD-1^+^ immune infiltrates and *PDCD1* gene expression offered the highest additional prognostic value for both OS and DFS, compared with traditionally used clinicopathological parameters (Table 4), including patient age, tumor size, tumor grade and lymph node status. These results, together with the finding that *CD274* or *PDCD1* may be used as independent prognostic markers, suggest a potential clinical application where mRNA levels may be used as a prognostic platform alone or combined with immunohistochemistry-based protein evaluation.

IPA of the 77-gene signature observed in the high *PDCD1* and *CD274* group in TNBC identified 12 genes associated with the canonical pathway “Communication between innate and adaptive immunity” (*IFNG, CCL5, CCR7, CD40LG, CD8A, CXCL10, HLA-DRB3, IGHA1, IGHD, IGHG1, IGHG3* and *IGHM*) and 4 genes associated with canonical pathway “Interferon signaling” (*MX1, STAT1, TAP1, IRF1*). The association with these genes is worthy of further study, as this may improve our understanding of TIL subsets; including macrophages, CD8^+^ T cells, B cells or plasma cells. As mentioned above, the inverse relationship between PD-L1 and MHC-I has been thought to be the mechanism underlying tumor escape from immune surveillance [88–91]. In the present study, we also demonstrated a strong positive association between PD-L1 and MHC-I at both the protein (Additional file 1: Figure S3) and mRNA (Additional file 1: Table S7) levels, which may explain why high PD-L1 expression in TNBC does not indicate a suppressive immune microenvironment or a poor prognosis. Furthermore, multiple interferon genes in the 77-gene list, including *IFNG, MX1, IRF1, IRF8, STAT1, CXCL9, CXCL10* and *CXCL11,* and particularly *IFNG*, have been demonstrated to be involved in immunoediting, and functionally related to PD-L1 and PD-1 [92–95]. IPA upstream regulator analysis further confirmed that *IFNG* was a significant common upregulator of these DEGs. Thus, their prognostic values were further investigated, and *IFNG* and certain related genes in the canonical pathway “Interferon signaling” were revealed to be of prognostic value in TNBC (Fig. 3h-k, Table 3), and were associated with PD-L1/PD-1 expression (Additional file 1: Tables S4, S5 and S7). This mechanism requires further detailed studies for confirmation. The antigen presentation may not have been impaired in the tumor in this case; rather, it may be accentuated.

Are PD-1^+^ immune cells, particularly CD8^+^ T cells, exhausted and therefore not functional? This question is commonly asked within the field of oncoimmunology. An increasing number of studies has suggested that PD-1 is not necessarily a marker of exhaustion, but also a marker of T cell activation and recent TCR signaling [109–114]. As it has also been reported as a marker of tumor reactivity [111], elevated numbers of PD-1^+^CD8^+^ T cells may also be reflective of higher numbers of tumor-specific T cells, which may be associated with improved patient outcomes. Along these lines, three previous studies have suggested that the immunosuppressive ATP ecto-nucleotidase CD39 is also an important marker of chronically stimulated and exhausted CD8^+^ T cells [114–116], is specific to both viral infections and the tumor microenvironment, and appears to be associated with tumor reactivity in the latter [116]. This suggests that PD-1 by itself does not define T cell exhaustion. Thus, it seems that expression of exhaustion-associated markers is associated with tumor reactivity and, in some cases, these cells may still be important for tumor control [46]. It should be noted that Odorizzi et al. [108] demonstrated that T cells can be differentiated to reach terminal exhaustion in the genetic absence of PD-1. In terms of defining T cell exhaustion, multiple reports have suggested the combination of PD-1 and transcription factor Eomesodermin (EOMES) might be more accurate [36, 117–120], and this warrants further study in a breast cancer setting. A recent breast cancer study also revealed that there is no significant reduction in cytokine production in PD-1^+^ T cells compared with PD-1^−^ T cells. Furthermore, PD-1^+^ T cells do not co-express LAG-3, TIM-3 or CTLA-4, which may suggest that PD-1^+^ T cells in breast cancer may not suffer from exhaustion, or at least support the argument that PD-1 expression alone does not indicate T cell exhaustion [46]. Overall, our results suggest that PD-1 expression on CD8^+^ T cells in TNBC does not preclude the ability of these cells to contribute to the control of tumor growth, since patients with more CD8^+^PD-1^+^ double positive immune subsets experienced significantly improved DFS (independent of overall CD8 densities). This suggests that patients with more CD8^+^PD-1^+^ T cells infiltrating tumor tissues experience a lower risk of recurrence.

Our data demonstrated that it is the CD8^+^PD-1^+^ immune subset, and not the CD8^−^PD-1^+^ immune subset, that offered prognostic value. Further studies to stratify the CD8^−^PD-1^+^ immune subsets in TNBC even further are warranted to characterize the immune microenvironment of TNBC. For example, are these primarily natural killer cells, CD4^+^ effector T cells, or regulatory T cells?

Clinical management options for TNBC remain limited, despite relatively high TIL numbers [16–18], PD-L1 expression [19–21] and tumor mutational burden [22, 23] compared with other subtypes, and multiple clinical trials have focused on targeting TNBC [16, 20, 22, 23, 121–124]. However, the outcome either remains sub-optimal or with conclusions still pending, regardless of which PD-1/PD-L1 monoclonal antibodies are used [7–15]. Our study may provide further insight to this field, as the results revealed that high expression of PD-1/PD-L1 pathways in TNBC was significantly associated with improved clinical outcomes. This suggests that the immune microenvironment in TNBC may not be as suppressed as in other tumors, such as non-small-cell lung carcinoma, melanoma and bladder cancer.

In conclusion, our study demonstrated that PD-1^+^ immune infiltrates, PD-L1 tumor cell expression and the expression of relevant genes are positively associated with an improved clinical outcome in TNBC. Furthermore, the prognostic values were independent of clinicopathological parameters. The mRNA levels of *PDCD1*, *CD274* and *IFNG* also represent measurable molecular prognostic markers. The function of the PD-1/PD-L1 pathway in the TNBC tumor immune microenvironment warrants further study, and may potentially provide alternative, effective novel targets for breast cancer immunotherapy in the near future. Finally, this may also help inform which combinations of strategies are most appropriate [125, 126].

## Additional file

Additional file 1: **Table S1.** Comparison of clinicopathological features of TNBC patients bearing high or low PD-L1 tumor cell expression and PD-1^+^ immune infiltrates. **Table S2.** Details of antibodies used for IHC labeling of TNBC sections. **Table S3.** IHC expression of immune markers in TNBCs. **Table S4.** Correlation between PD-L1 tumor cell expression, PD-1^+^ immune infiltrates and RNA expression of the relevant genes in TNBCs. **Table S5.** Correlation between PD-1^+^ immune infiltrates and the RNA expression of the relevant genes in TNBCs. **Table S6.** Analysis of *PDCD1* and *CD274* expression levels and survival outcomes in TNBC using data from the European Genome-Phenome Archive. *n* = 320. **Table S7.** Correlation between *CD274*, *PDCD1* and HLA mRNA expression in triple negative breast cancer. **Figure S1.** TNBC with high *PDCD1* and high *CD274* expression exhibit distinct gene expression signatures. Heat map of the 77 significantly differentially-expressed genes (*P* < 0.05) showing specific expression profiles in high and low *PDCD1* and *CD274* expression, clustered using Euclidean distances on the z scores computed from the log10 transformed counts. The heat map is colored using z scores with the highest expression in yellow and the lowest expression in blue. *PDCD1* (encoding PD-1), *CD274* (encoding PD-L1). **Figure S2.** TNBC with both high *PDCD1* and high *CD274* expression show a trend for improved survival in a public dataset from TCGA. From publicly available TNBC dataset from TCGA, Kaplan-Meier analysis of OS outcomes in women with high *PDCD1* and high *CD274* expression compared with the rest of the cases in the cohort (*n* = 89). TNBC, Triple negative breast cancer; TCGA, The Cancer Genome Atlas. The trend is observed but the statistical significance is not achieved probably due to the small sample size in this public dataset. **Figure S3.** Flow cytometry analysis demonstrated the correlation between PD-L1 and MHC-I on multiple human TNBC cell lines. PDL1, programmed cell death ligand 1; MHC-I, major histocompatibility complex class I (HLAABC). **Figure S4.** Expression levels of a panel of 5 genes from *IFN* signaling define two groups of TNBC patients. Unsupervised hierarchical clustering using Euclidean distance revealed the existence of two TNBC patient clusters (red and green) based on expression intensity of the 5 genes listed. The heat map is colored by the log10 normalized counts with the highest expression in red and the lowest expression in blue. **Figure S5.** Scoring of PD-1^+^ immune infiltrates data on TMA can be validated with whole section scoring. (A) Manual scoring on whole slide sections shows that TNBCs bearing high PD-1^+^ immune infiltrates (tissue microarray analyses) harbored significantly higher PD-1^+^ immune infiltrates. (B) Manual scoring on whole slide sections shows significant correlation with the scoring done on tissue microarray. (DOCX 413 kb)
