# Supplementary material for: Prognostic value of CD8 + PD-1+ immune infiltrates and PDCD1 gene expression in triple negative breast cancer
Source: J Immunother Cancer. 2019 Feb 6;7:34. doi: 10.1186/s40425-019-0499-y (PMC6366051; doi:10.1186/s40425-019-0499-y)

**Additional file 1**

**Table S1**. Comparison of clinicopathological features of TNBC patients bearing high or low PD-L1 tumor cell expression and PD-1^+^ immune infiltrates.

|  | **PD-L1** | | | **PD-1** | | |
| --- | --- | --- | --- | --- | --- | --- |
| **Factors** | Negative | Positive | P-value | Negative | Positive | P-value |
| **Age^ (years)** | 55.4 (12.7) | 56.9 (9.9) | 0.3258 | 56.8 (12.4) | 54.8 (11.7) | 0.1902 |
| **Tumor size (mm)** |  |  | 0.5193 |  |  | 0.3760 |
| $\leq$20 | 50 | 15 |  | 29 | 31 |  |
| >20 | 141 | 54 |  | 103 | 84 |  |
| **Tumor grade** |  |  | 0.1773 |  |  | 1.0000 |
| 1/2 | 34 | 7 |  | 19 | 16 |  |
| 3 | 158 | 62 |  | 112 | 100 |  |
| **Lymph node stage (pN)** |  |  | 0.5308 |  |  | 0.3402 |
| 0 | 81 | 31 |  | 61 | 44 |  |
| 1 | 37 | 11 |  | 21 | 23 |  |
| 2 | 22 | 4 |  | 13 | 12 |  |
| 3 | 12 | 2 |  | 11 | 4 |  |
| **Lymphovascular**  **invasion** |  |  | 0.1898 |  |  | 0.0337* |
| Absent | 119 | 50 |  | 77 | 83 |  |
| Present | 74 | 20 |  | 56 | 33 |  |
| **Ethinicity** |  |  | 0.4664 |  |  | 0.4166 |
| Chinese | 135 | 54 |  | 94 | 85 |  |
| Indian | 9 | 1 |  | 5 | 5 |  |
| Malay | 10 | 3 |  | 6 | 7 |  |
| Other | 11 | 7 |  | 5 | 11 |  |

*Statistically significant. PD-1, programmed cell death protein-1; PD-L1, programmed cell death ligand 1

^Age is presented as mean (standard deviation)

**Table S2**. Details of antibodies used for IHC labeling of TNBC sections.

| **Antibody** | **Clone** | **Dilution** | **Source** | **Labeling Pattern** |
| --- | --- | --- | --- | --- |
| ER (IHC) | SP1 | 1:50 | Thermo Scientific Lab Vision RM 9101-S | Tumor cells, Nuclear |
| PR (IHC) | SP2 | 1:200 | Thermo Scientific Lab Vision RM9102-S | Tumor cells, Nuclear |
| HER2 (IHC) | SP3 | 1:200 | Thermo Scientific Lab Vision RM9103-S | Tumor cells, Membranous |
| CK14 (IHC) | LL002 | 1:20 | Leica Novocastra NCL-L-LL002 | Tumor cells, Cytoplasmic |
| EGFR (IHC) | E30 | 1:50 | Dako M7239 | Tumor cells, Membranous |
| CK, high molecular weight, 34βE12 (IHC) | 34βE12 | 1:200 | Dako M0630 | Tumor cells, Cytoplasmic |
| PD-1 (IHC, IF) | NAT105 | 1:100 | Cell Marque 315M-96 | Immune cells, Cytoplasmic |
| PD-L1 (IHC) | E1L3N | 1:600 | Cell Signaling mAb #13684 | Tumor cells, Membranous |
| CD8 (IF) | 1A5 | 1:30 | Leica Novocastra NCL-CD8-295 | Immune cells, Membranous and/or cytoplasmic |

IHC, immunohistochemistry; IF, immunofluorescence, ER, estrogen receptor; PR, progesterone receptor; HER2, c-erbB2; CK, cytokeratin; EGFR, epidermal growth factor receptor; PD-1, programmed cell death protein-1; PD-L1, programmed cell death ligand 1. Thermo Scientific Lab Vision (Thermo Fisher Lab Vision, Fremont, CA, USA), Leica Novocastra (Leica Biosystems Newcastle, UK), Dako (Dako North America, USA), Cell Marque (Cell Marque, Rocklin, CA, USA).

**Table S3**. IHC expression of immune markers in TNBCs.

| **Marker** | **N (samples tested)** | **Intra-tumoral** | |
| --- | --- | --- | --- |
| **PD-1** | 247 | IHC Median cut-off = 1 count per 1mm core | |
|  |  | Number of samples | % of samples |
|  | *High* | 115 | 46.6 |
|  | *Low* | 132 | 53.4 |
| **PD-L1** | 260 | IHC Median cut-off = 1% tumor proportion score | |
|  |  | Number of samples | % of samples |
|  | *High* | 69 | 26.5 |
|  | *Low* | 191 | 73.5 |

IHC, immunohistochemistry; PD-1, programmed cell death protein-1; PD-L1, programmed cell death ligand 1.

**Table S4.** Correlation between PD-L1 tumor cell expression, PD-1^+^ immune infiltrates and RNA expression of the relevant genes in TNBCs.

|  | PD-1 | *PDCD1* | *CD274* | *HLA-A* | *HLA-B* | *HLA-C* | *IFNG* |
| --- | --- | --- | --- | --- | --- | --- | --- |
| PD-L1 | R= 0.303  P< 0.0001* | R= 0.213  P< 0.002* | R= 0.411  P< 0.0001* | R= 0.262  P= 0.0001* | R= 0.279  P< 0.0001* | R= 0.225  P= 0.001* | R= 0.300  P< 0.0001* |

*Statistically significant. PD-1, programmed cell death protein-1; PD-L1, programmed cell death ligand 1.

**Table S5.** Correlation between PD-1^+^ immune infiltrates and the RNA expression of the relevant genes in TNBCs.

|  | *PDCD1* | *CD274* | *HLA-A* | *HLA-B* | *HLA-C* | *IFNG* |
| --- | --- | --- | --- | --- | --- | --- |
| PD-1 | R= 0.276  P< 0.0001* | R= 0.321  P< 0.0001* | R= 0.277  P= 0.0001* | R= 0.284  P< 0.0001* | R= 0.181  P= 0.0075* | R= 0.254  P= 0.0002* |

*Statistically significant

**Table S6.** Analysis of *PDCD1* and *CD274* expression levels and survival outcomes in TNBC using data from the European Genome-Phenome Archive. n=320.

| **Biomarkers** | **HR** | **95% CI** | **P-value** |
| --- | --- | --- | --- |
| *OS* | | | |
| ***PDCD1* expression**  (every 1 unit increment) | 0.55 | 0.26 - 1.12 | 0.086 |
| ***CD274* expression**  (every 1 unit increment) | 0.77 | 0.55-1.08 | 0.121 |
| *DFS* | | | |
| ***PDCD1* expression**  (every 1 unit increment) | 0.38 | 0.15 - 0.94 | 0.027* |
| ***CD274* expression**  (every 1 unit increment) | 0.63 | 0.42-0.96 | 0.026* |

*Statistically significant.

**Table S7.** Correlation between *CD274*, *PDCD1* and HLA mRNA expression in triple negative breast cancer.

|  | *PDCD1* | *HLA-A* | *HLA-B* | *HLA-C* | *IFNG* |
| --- | --- | --- | --- | --- | --- |
| *CD274* | R= 0.499  < 0.0001* | R= 0.543  < 0.0001* | R= 0.523  P< 0.0001* | R= 0.418  P= 0.001* | R= 0.553  P< 0.0001* |

*Statistically significant.

**Figure S1. TNBC with high *PDCD1* and high *CD274* expression exhibit distinct gene expression signatures.** Heat map of the 77 significantly differentially-expressed genes (P<0.05) showing specific expression profiles in high and low *PDCD1* and *CD274* expression, clustered using Euclidean distances on the z scores computed from the log10 transformed counts. The heat map is colored using z scores with the highest expression in yellow and the lowest expression in blue. *PDCD1* (encoding PD-1), *CD274* (encoding PD-L1).


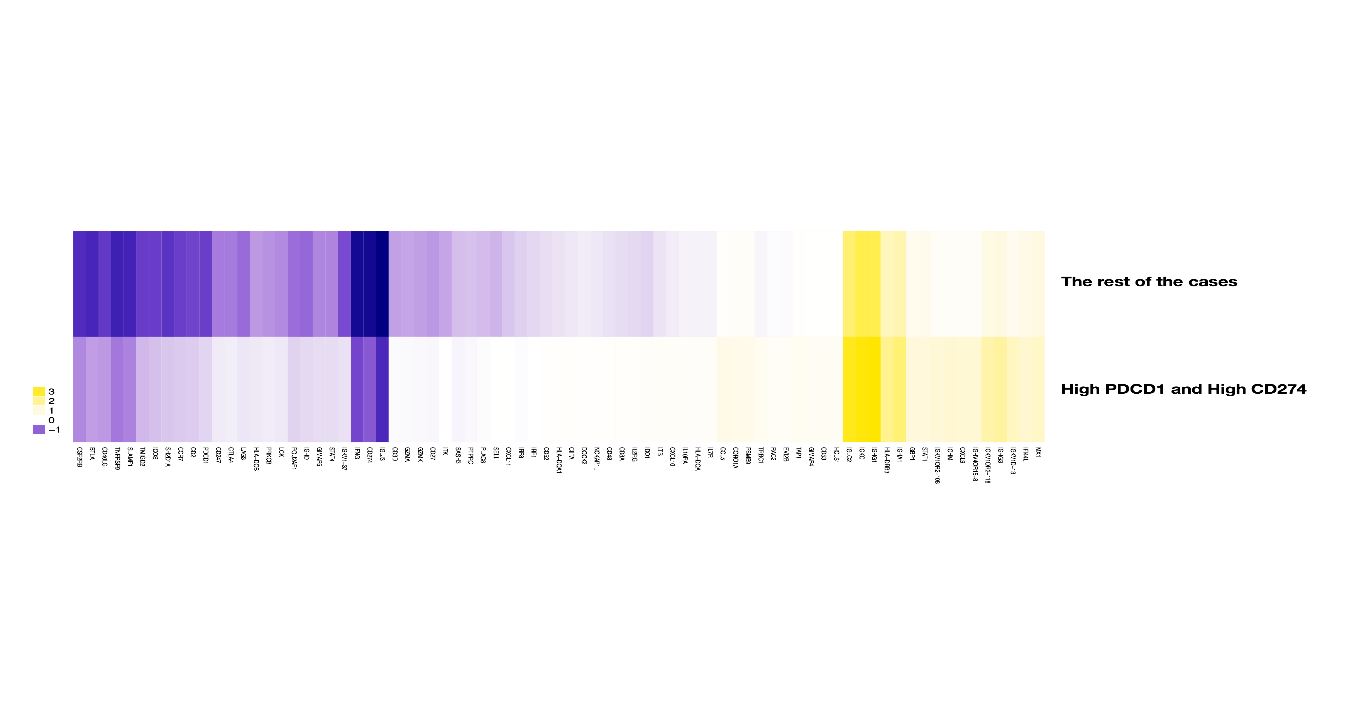


**Figure S2**. **TNBC with both high *PDCD1* and high *CD274* expression show a trend for improved survival** **in a public dataset from TCGA.** From publicly available TNBC dataset from TCGA, Kaplan-Meier analysis of OS outcomes in women with high *PDCD1* and high *CD274* expression compared with the rest of the cases in the cohort (n=89). TNBC, Triple negative breast cancer; TCGA, The Cancer Genome Atlas. The trend is observed but the statistical significance is not achieved probably due to the small sample size in this public dataset.

**
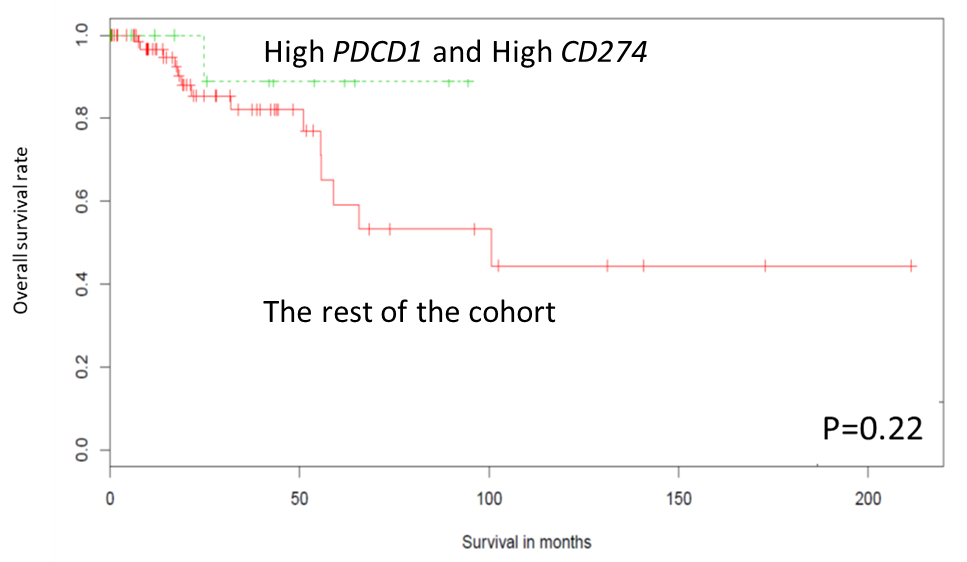
**

**Figure S3**. Flow cytometry analysis demonstrated the correlation between PD-L1 and MHC-I on multiple human TNBC cell lines. PDL1, programmed cell death ligand 1; MHC-I, major histocompatibility complex class I (HLAABC).

**Figure S4**. **Expression levels of a panel of 5 genes from *IFN* signaling define two groups of TNBC patients.** Unsupervised hierarchical clustering using Euclidean distance revealed the existence of two TNBC patient clusters (red and green) based on expression intensity of the 5 genes listed. The heat map is colored by the log10 normalized counts with the highest expression in red and the lowest expression in blue.


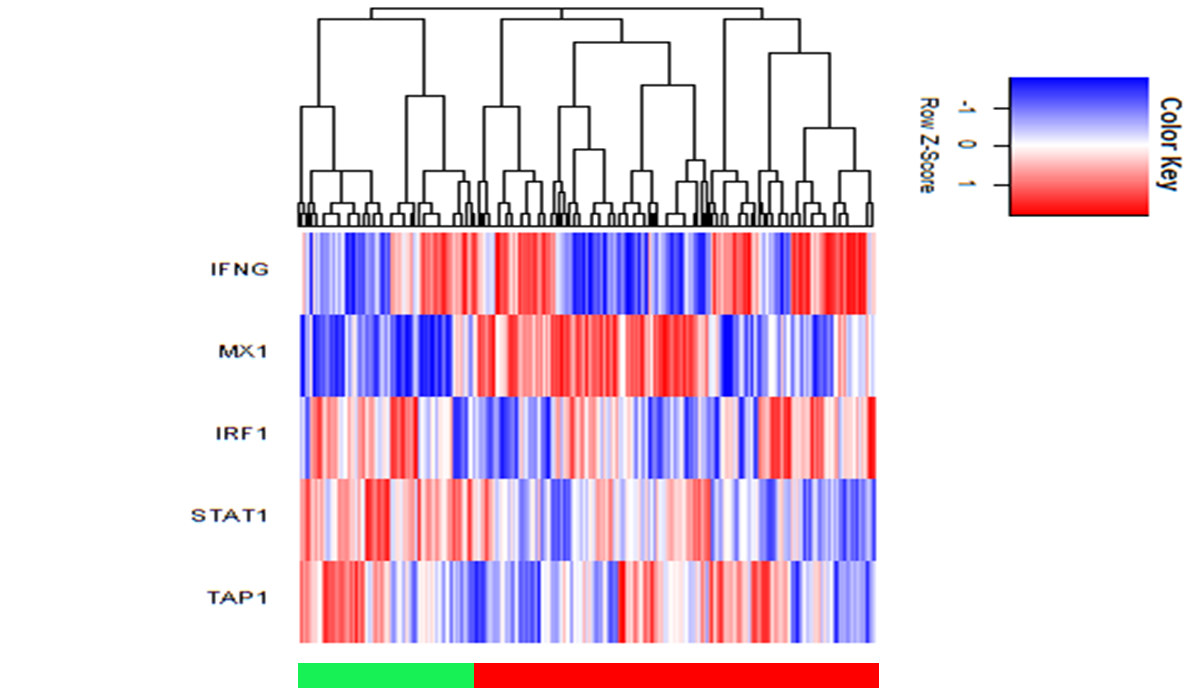


**Figure S5. Scoring of PD-1^+^ immune infiltrates data on TMA can be validated with whole section scoring.** (A) Manual scoring on whole slide sections shows that TNBCs bearing high PD-1^+^ immune infiltrates (tissue microarray analyses) harbored significantly higher PD-1^+^ immune infiltrates. (B) Manual scoring on whole slide sections shows significant correlation with the scoring done on tissue microarray.


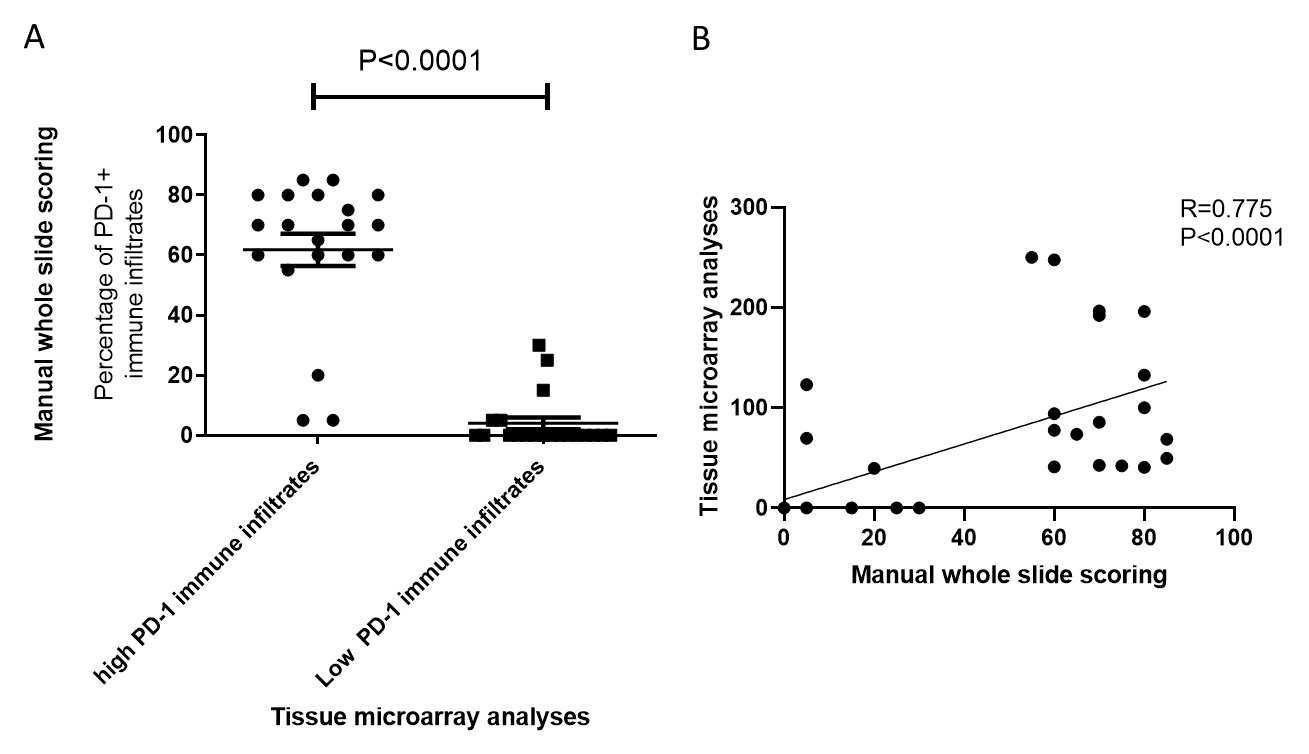

Supplement: Supplementary file 1 — Table S1. Comparison of clinicopathological features of TNBC patients bearing high or low PD-L1 tumor cell expression and PD-1+ immune infiltrates. Table S2. Details of antibodies used for IHC labeling of TNBC sections. Table S3. IHC expression of immune markers in TNBCs. Table S4. Correlation between PD-L1 tumor cell expression, PD-1+ immune infiltrates and RNA expression of the relevant genes in TNBCs. Table S5. Correlation between PD-1+ immune infiltrates and the RNA expression of the relevant genes in TNBCs. Table S6. Analysis of PDCD1 and CD274 expression levels and survival outcomes in TNBC using data from the European Genome-Phenome Archive. n = 320. Table S7. Correlation between CD274, PDCD1 and HLA mRNA expression in triple negative breast cancer. Figure S1. TNBC with high PDCD1 and high CD274 expression exhibit distinct gene expression signatures. Heat map of the 77 significantly differentially-expressed genes (P < 0.05) showing specific expression profiles in high and low PDCD1 and CD274 expression, clustered using Euclidean distances on the z scores computed from the log10 transformed counts. The heat map is colored using z scores with the highest expression in yellow and the lowest expression in blue. PDCD1 (encoding PD-1), CD274 (encoding PD-L1). Figure S2. TNBC with both high PDCD1 and high CD274 expression show a trend for improved survival in a public dataset from TCGA. From publicly available TNBC dataset from TCGA, Kaplan-Meier analysis of OS outcomes in women with high PDCD1 and high CD274 expression compared with the rest of the cases in the cohort (n = 89). TNBC, Triple negative breast cancer; TCGA, The Cancer Genome Atlas. The trend is observed but the statistical significance is not achieved probably due to the small sample size in this public dataset. Figure S3. Flow cytometry analysis demonstrated the correlation between PD-L1 and MHC-I on multiple human TNBC cell lines. PDL1, programmed cell death ligand 1; MHC-I, major histocompatibility [file 40425_2019_499_MOESM1_ESM.docx]
